# Drought-tolerant fungal microbes, *Aspergillus oryzae* and *Aspergillus fumigatus*, elevate physiohormonal and antioxidant responses of maize under drought stress

**DOI:** 10.3389/fmicb.2024.1488639

**Published:** 2024-11-28

**Authors:** Kiran Niaz, Mamoona Rauf, Muhammad Arif, Muhammad Hamayun, Humaira Gul, Abeer Hashem, Elsayed Fathi Abd_Allah, Qiang-Sheng Wu

**Affiliations:** ^1^Department of Botany, Abdul Wali Khan University Mardan, Mardan, Pakistan; ^2^Department of Biotechnology, Abdul Wali Khan University Mardan, Mardan, Pakistan; ^3^Botany and Microbiology Department, College of Science, King Saud University, Riyadh, Saudi Arabia; ^4^Plant Production Department, College of Food and Agricultural Sciences, King Saud University, Riyadh, Saudi Arabia; ^5^College of Horticulture and Gardening, Yangtze University, Jingzhou, China

**Keywords:** beneficial fungal microbes, plant–microbe interaction, drought stress, phytohormones, metabolites, maize

## Abstract

**Introduction:**

Temporary and extended drought stress accelerates phytohormones and reactive oxygen species (ROS) in plants, however, the fate of the plants under stress is mostly determined by the metabolic and molecular reprogramming, which can be modulated by the application of habitat-adapted fungi that triggers resistance to stress upon symbiotic association.

**Methods:**

The present research exhibited the exploitation of the newly isolated, drought habitat-adapted fungal endophytic consortium of SAB (*Aspergillus oryzae*) and CBW (*Aspergillus fumigatus*), on maize under drought stress. SAB and CBW primarily hosted the root tissues of *Conyza bonariensis* L., which have not been reported earlier, and sufficiently produced growth-promoting metabolites and antioxidants.

**Results:**

SAB and CBW adeptly inhabited the maize roots. They promoted biomass, primary metabolites, osmolytes (protein, sugar, lipids, proline, phenolics, flavonoids), and IAA production while reducing tannins, ABA, and H_2_O_2_ contents and increasing antioxidant enzyme activities. In addition, the enhanced adventitious root development at the root/stem interface, and elongated main root development optimum stomatal activity of SAB- and CBW-inoculated maize plants were observed under drought stress. SAB and CBW modulated the expression of the *ZmBSK1*, *ZmAPX*, and *ZmCAT1* genes in the maize shoot and root tissues under drought stress vs. control, signifying an essential regulatory function for SAB/CBW-induced drought stress tolerance via phytohormonal signaling pathway leading to the antioxidant upregulation.

**Discussion:**

These findings imply that the exogenous administration of the SAB/CBW consortium might be a rather efficient strategy that contributes to optimizing the physio-hormonal attributes and antioxidant potential to alleviate the drought stress in maize.

## Graphical abstract

Graphical abstract depicting the potential role of habitat-adapted fungal endophytes SAB and CBW cultivated maize resilience to drought by enhancing the growth, and stress tolerance, via brassinosteroid regulation, metabolic induction, elevation of osmolytes, antioxidant activation, and stomatal activity optimization.

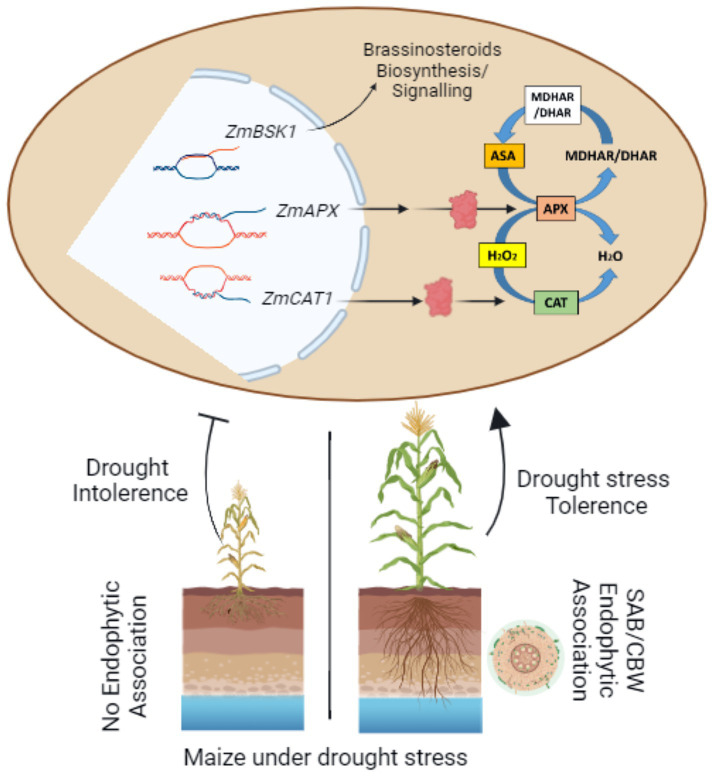

## 1 Introduction

Drought directly and indirectly impacts agricultural output, concerns global food security, and threatens the sustainability of crop farming organizations because of rapidly changing meteorological and oceanographic circumstances (Verma et al., 2021). It is anticipated that approximately 30% of water resources will drop if the scenario continues, and drought areas will certainly double by 2050. Thus, it is critical to explore aspects that subsidize improved drought resistance in plants including maize (*Zea mays*) (Siddique et al., 2022). Drought stress is a major barrier to agricultural productivity in semiarid and arid global areas. Plants must quickly adjust to water scarcity, impacting biological functions at the plant level. Earth’s surface is divided into hyperarid, arid, semiarid, and dry subhumid zones (UN, 2010). Meteorological drought impacts 21% of the world’s land, with 13% experiencing moderate-to-severe conditions, posing a significant global hazard to agricultural output and plant growth. Maize is a starch-rich crop used to synthesize ethanol and is commonly utilized in biofuel production. It is attractive because of their ability to biodegradability, decrease greenhouse gas emissions, and clean bursting, thus improving energy security (Sikiru et al., 2024).

As sessile organisms, plants are exposed to various ecological strains that contribute to reduced quality traits of plants, such as height, stem girth, and leaf size, leading to decreased water content, lowered leaf water potential, turgor loss, cell enlargement, and photosynthetic pigments (Damberg and AghaKouchak, 2014). Stress-tolerant plant varieties have evolved defensive mechanisms against stressors, controlling growth and performance. Stress-reduction strategies involve hyperactivating ROS-scavenging machinery, increasing antioxidant enzyme activities, and activating stress-related genes in stress-tolerant genotypes, thereby increasing plant resistance to drought stress and ROS detoxification that deteriorate the biomolecules (protein, carbohydrates, and DNA/RNA). The term “xerophytic” refers to plants that can withstand drought by using strategies, including (i) osmolyte synthesis and accumulation to regulate turgor pressure and prevent structural membrane injury; (ii) regulating ionic equilibrium and balancing; (iii) modulating phytohormone biosynthesis and signaling, (iv) metabolic reshuffling related to growth promotion and stress mitigation; and (v) genetic adaptation through moderating the expression of genes that drive stress perception and cell signaling, as well as initiating the stress tolerance response (Yang et al., 2021).

Nevertheless, a lack of adaptability makes several plant species susceptible to stresses that harm plant vigor, growth, and development.

Apart from the physiological, biochemical, and metabolic aspects of adaptations, the adaptive underlying molecular mechanisms for drought stress tolerance remain unclear despite extensive efforts. Therefore, researchers are developing highly resistant crop varieties to address drought resistance, but efforts are limited due to low heritability and complex genetic reactions due to abiotic stresses (Eke et al., 2019). Prior research has revealed that protein kinases, for example, calcium−/calmodulin-dependent protein kinase (CCaMK) and mitogen-activated protein kinase (MAPK), favorably control plant responses to drought stress by triggering antioxidant defense systems (Ni et al., 2019). Research on drought tolerance in plants using omics reveals gene expression manipulation, post-transcriptional alterations, and translational modifications, but further investigation is required to comprehend molecular bases.

In agricultural applications, the alternative strategy is the direct application of exogenous chemicals as a crop supplement. The substances supplied are generally intermediate metabolites of sugars, sugar alcohols, and amino acids, like *γ*-aminobutyric acid (Hayat et al., 2023), trehalose and sorbitol (Theerakulpisut and Gunnula, 2012), ascorbic acid (Khazaei and Estaji, 2020), 6-benzylaminopurine (Rezaei et al., 2020), methyl jasmonate and salicylic acid (Tayyab et al., 2020), and abscisic acid (Awan et al., 2021). As Zhang et al. (2022) discovered that exogenous brassinosteroids improve drought stress tolerance, promote root growth, and increase maize yield. Therefore, one of the main rational behind this investigation was the use of chemical-based drought mitigation approaches is costly and time-consuming. Thus, instead of using synthetic brassinosteroids in maize for improving drought stress tolerance, the environment-friendly strategy is adapted by exploitation of endophytic fungi as bioengineers to explore their role of drought tolerance induction through brassinosteroids biosynthesis, which has not been studied yet.

Brassinosteroids are a class of biomolecules (polyhydroxylated steroidal phytohormones) that regulate the division, elongation, and differentiation of a wide range of cell types. Plant scientists have become interested in recent studies due to their adaptability to various environmental challenges. On the other hand, scientists around the world have recently utilized growth-promoting beneficial microbes from harsh environments as an effective biological method for improving stress resistance in plants as it improves ecological adaption to severe environments such as rice, luffa, moringa, wheat, tomato, and okra (Aziz et al., 2021; Rauf et al., 2022; Rahman et al., 2023; Gul et al., 2023).

However, research on exploring the mechanism and maize response for drought resistance induction by endophytic fungal consortiums via brassinosteroids biosynthesis is still insufficient. To date, the isolated endophytes from harsh habitats have been known to lessen drought stress in a variety of crops by modulating various morphological variations and physiological mechanisms. For example, *Cladosporium cladosporioides* isolated from wild tobacco amplified drought resistance by modifiable antioxidants and osmolytes in *Nicotiana benthamiana* plants (Dastogeer et al., 2018); osmotic stress-tolerant *Paraphoma* sp., *Entomophaga chlamydospora*, and *Cladosporium oxysporum* isolated from *Helianthemum scoparium* induced drought stress tolerance in the host (*Helianthemum scoparium*) and non-host (*Glycyrrhiza uralensis* and *Zea mays*) (Li et al., 2019); consortia of *Microdochium majus*, *Aspergillus aculeatus*, and *Meyerozyma guilliermondii* isolated from *Carthamus oxyacantha* induced tolerance in *Moringa oleifera* under drought stress (Javed et al., 2022). Similarly, the *Fusarium proliferatum* obtained from the roots of *Rhazya stricta* increases the drought resistance of sunflowers (Seema et al., 2023). In the current study, *Conyza bonariensis* L. Cronquist (hairy fleabane), a highly competitive and proliferative weed from *Asteraceae* family (Kaspary et al., 2017) mainly growing well in arid regions, has been focused for its multitrees-tolerant responses having resistance to drought, salt, and herbicides (inhibitors of 5-enolpyruvylshikimate-3-phosphate synthase, acetolactate synthase, and synthetic auxins (reviewed by Kaspary et al., 2024)). Therefore, the present investigation was based on the hypothesis that drought-resistant endophytic fungi residing inside host from the drought environment may enhance the growth of maize and alleviate the drought stress.

Hence, the present investigation proposed the isolation and characterization of the drought-tolerant endophytic fungi from arid environments to explore their role in drought stress tolerance induction of maize via modulation of physiochemical properties, antioxidant potential, and phytohormonal contents.

## 2 Methods

### 2.1 Fungal endophytes isolation, identification, and characterization

**Figure 1:**
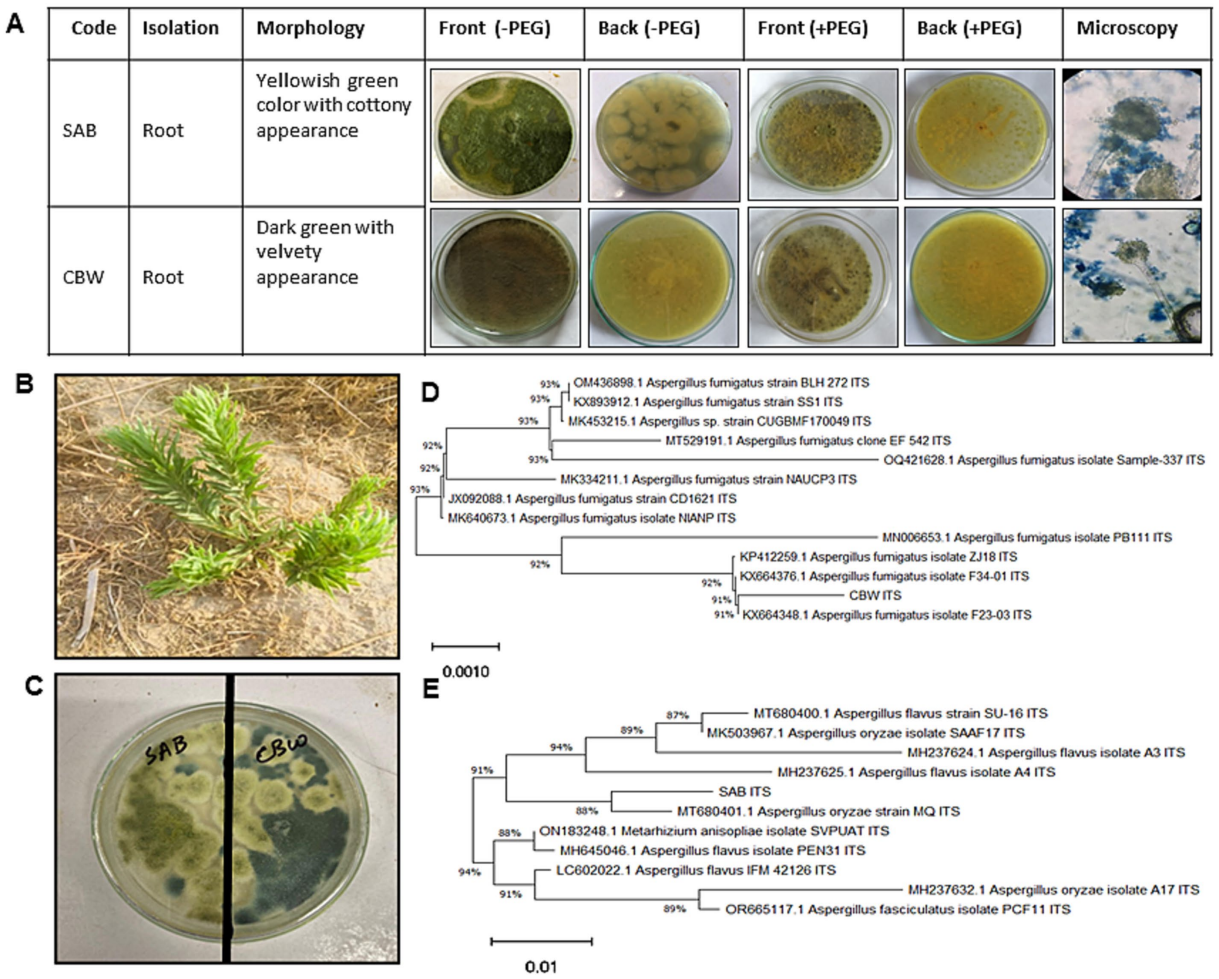
Morphologic description of SAB and CBW. **(A)** Phenotypic features SAB and CBW, **(B)** *C. bonariensis*, **(C)** dual-culture plate assay showing growth compatibility of SAB and CBW isolates, **(D)** phylogenetic analysis of CBW, and **(E)** phylogenetic analysis of SAB.

For endophytic fungal isolation, the selected plant (vegetative stage), as shown in Figure 1B, was uprooted from arid environments in Hazro, Attock (N33°54′35.64″, E72°29′30.48″), Pakistan, brought to the Plant Microbe Interaction Lab, Abdul Wali Khan University Mardan, Pakistan, and identified as *Conyza bonariensis* L. Cronquist (hairy fleabane) previously reported as drought-tolerant weed plant having resistance to herbicides, including inhibitors of 5-enolpyruvylshikimate-3-phosphate synthase, acetolactate synthase, and synthetic auxins (reviewed by Kaspary et al., 2024). The collected plant material was segmented, and root tissues were tap-washed, sterilized with 70% ethanol for 2 min, and rinsed with the dH_2_O to remove the ethanol residues. The sterilized root tissues were shared into segments, and 10 parts were placed per plate containing hagem medium. The arising fungal colony was inoculated on potato dextrose agar (PDA) medium, inoculated at 25°C for 7 days, and arising colonies were taken into consideration to achieve respective pure culture by subsequent individual subculturing on PDA medium. Similarly, the fungal morphological features were observed under the microscope at the magnification of 40× and 100×. Additionally, fungal colonies were stained and intensified by Lactophenol Cotton Blue reagent following the previously described method (Ali et al., 2022). Extracted genomic DNA (20 ng) was extracted, and the rDNA gene (ITS region) was subjected to amplification by PCR by preparing a total 30 μL reaction of the mixture using ITS1 primer (TCCGTAGGTGAACCTGCGG), and ITS4 primer (TCCTCCGCTTATTGATATGC) (White et al., 1990) *and* sequenced by BGI Co., Ltd. (Shenzhen, China), as described earlier (Javed et al., 2022). The sequences were subjected to the NCBI^1^ for homology search and phylogenetic trees of the neighbor-joining (NJ) were constructed by using the MEGA X. The sequences were deposited to NCBI GenBank under accession numbers, PP892795 and PP859235.

### 2.2 Screening of fungal endophytes in PEG (polyethylene glycol) containing Czapek medium

**Figure 2:**
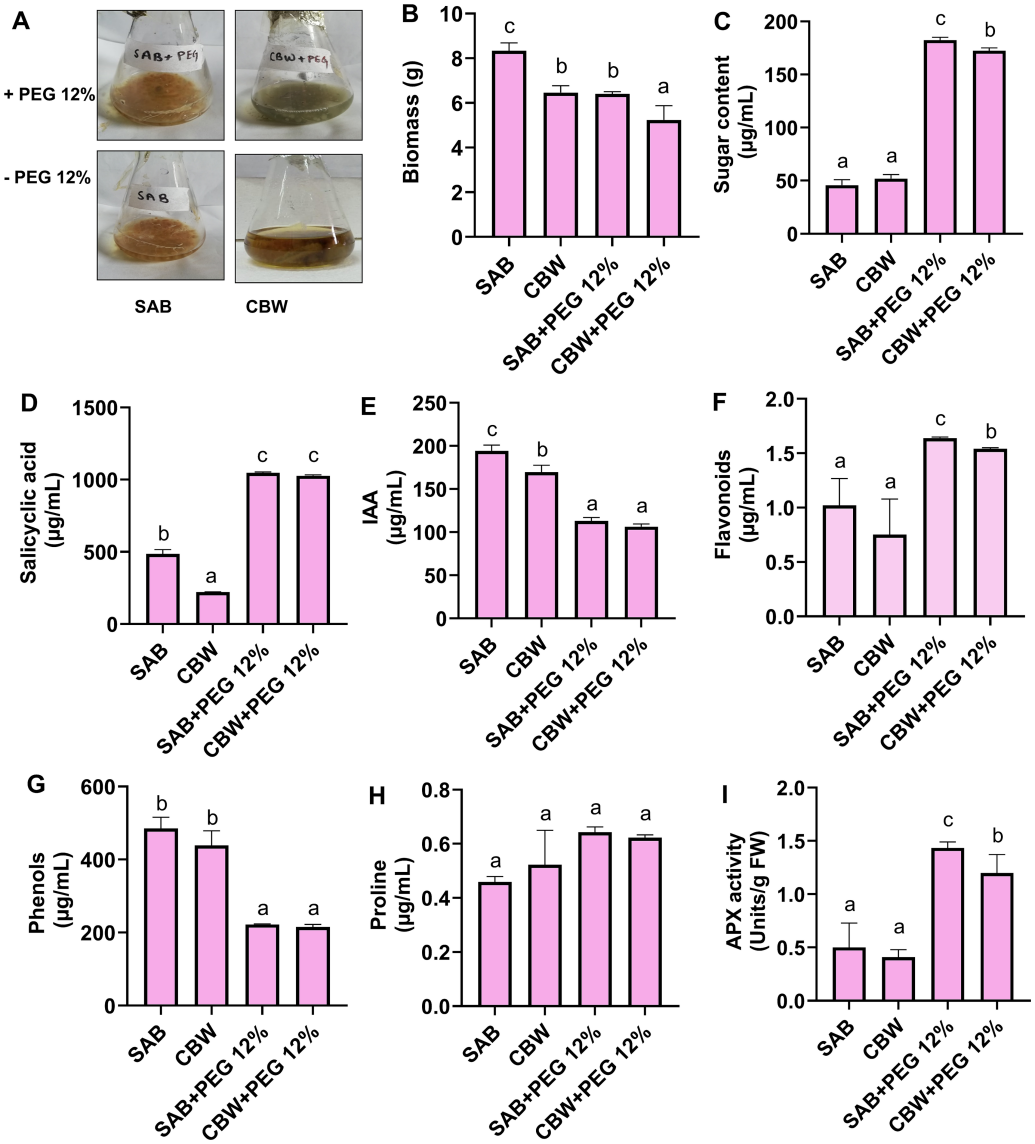
*In Vitro* growth response of SAB and CBW upon 12% PEG supplementation. **(A)** Growth of fungal isolates in PEG 12%, **(B)** biomass production, **(C)** total soluble sugars, **(D)** salicylic acid, **(E)** IAA, **(F)** flavonoid, **(G)** phenol, **(H)** proline, and **(I)** APX activity. Numerical data signify means ± SE presented as biologically distinct replicates with significant letters at *p ≤* 0.05 via a Duncan multiple range test (DMRT).

A total of 12 distinctly purified strains were selected for the preliminary screening upon drought stress induced by various concentrations of PEG-6000 (0, 6, and 12%), previously reported as osmoticum to induce the osmotic pressure in fungal cells in Czapek medium (50 mL) (Javed et al., 2022). The flasks were kept in the shaking incubator at 120 rpm for 7 days at 30°C, and the growth rate was checked. Similarly, after 7 days, the fresh biomass/flask of each culture (0 and 6, and 12% PEG) was observed. Among all endophytic fungi, only two strains (SAB and CBW) ably produced biomass under 12% PEG-induced drought stress, hence selected as drought stress-resistant fungal endophytes (Figure 2A). Drought stress-tolerant active biomass of fungal mycelia and fungal culture filtrate was separated for further investigation under this study.

### 2.3 Evaluation of the compatibility/antagonistic response using the dual-culture plant approach

In a dual-culture experiment as described earlier (Rahman et al., 2023), the co-cultivation assay on the PDA nutritional medium plate was used to assess the antagonistic response and compatibility of the SAB and CBW, isolates against one another. SAB and CBW samples, containing 1 × 10^7^ spores mL^−1^, were injected with a total of 20 μL in the center of PDA plates. It was solely SAB and CBW in the control cultures. Incubation was held at 26 ± 2°C, and the experiment was run in triplicate.

### 2.4 Endophytic consortia-inoculum preparation

Fungal inoculum preparation was done using Czapek medium (100 mL). The fungal growth was observed after 7 days of incubation in a shaking incubator at 120 rpm and 30°C. After the incubation period, the fresh fungal biomass of the spore suspension (∼5 × 10^7^ spores/mL) was preserved for each endophytic culture.

### 2.5 Determination of hormones, metabolites, osmolyte, and antioxidants in endophytic fungal culture filtrate

Indole-3-acetic acid and salicylic acid, proteins, sugars, lipids, phenolics, flavonoids, proline, and antioxidants were measured in fungal culture filtrates. IAA estimation was performed on the cultural filtrate using the Salkowski reagent method (Benizri et al., 1998). Optical density was recorded at 540 nm against a blank, the standard curve was created by IAA (10–100 μg/mL), and salicylic acid (SA) was estimated following the procedure indicated previously (Warrier et al., 2013).

The total soluble sugars were determined as defined earlier (Mohammadkhani and Heidari, 2008), Estimating the proline content was done with the standard protocol of Bates et al. (1973), and total proteins were measured using the method of Lowry et al. (1951), and total lipids were quantified as mentioned earlier (Vogel, 1956). From the culture filtrate, flavonoids and phenols were estimated as mentioned previously (Khatiwora et al., 2012). In brief, 0.2 mL of culture supernatant was mixed with 2 mL of 7.5% Na_2_CO_3_ and 0.8 mL of Folin–Ciocalteu reagent. The mixture was diluted to seven volumes with dH_2_O and kept for 2 h in the dark. The catechol (1–10 mg/mL) was used as standard, and optical density was recorded at 650 nm. Additionally, peroxidase activity in the culture filtrate was quantified following the earlier procedure (Malik and Singh, 1980).

### 2.6 Application of fungal isolates SAB and CBW to maize under drought stress

#### 2.6.1 *In vitro* drought tolerance bioassay of maize in pot experiments

To assess the drought stress ameliorating effect of endophytic inoculation by SAB and CBW on maize, the certified seeds of the Gohar-19 variety were obtained from the National Agricultural Research Centre (NARC), Islamabad, Pakistan. Maize variety, Gohar-19, is one of Pakistan’s CIMMYT-developed maize varieties that is short duration and can be harvested quickly to rotate land for the next crop. They can also be grown in the main and off season, which makes them suitable for many different cropping systems. However, its drought stress response was unexplored. Therefore, current study was focused on maize (Gohar-19).

Healthy seeds were rinsed thrice with dH_2_O, sterilized with 70% ethanol, washed thrice, and grown in the soil. One commonly used method for *in vitro* screening of drought tolerance in plants involves inducing stress using osmotic agents like PEG-6000 as it has a high molecular weight, inert, and non-toxic and simulates drought conditions without any harmful effects on cells (Jiang et al., 1995). Previous reports have shown that monocot plants such as maize and wheat tolerance to PEG-induced drought stress vary depending on the concentration, variety, and growth stage, and several reports have shown that various maize varieties differentially proved sensitive at germination and seedling stages to PEG-induced drought stress with a significant reduction in germination rate, root elongation, and shoot development at a concentration above 10% PEG-6000 (at 10, 20, 30, 40, and 50%) (Elizabeth Mustamu et al., 2023), and wheat seedlings in soil system (Nelson and Oliver, 2017). Therefore, PEG-mediated drought-induction plant bioassay was carried out at the level of 12% PEG-6000.

#### 2.6.2 Drought stress induction treatment plant bioassay

For fungal inoculation, the active biomass (3 g/500 g per pot) was supplemented to the soil. Experiment was conducted thrice, and experimental set-up was completely randomized design (CRD) comprising at least 12 plastic pots (8.5 cm diameter; 12.5 cm depth) for each treatment filled with sterile sandy loam soil (Table 1). The water content of soil was adjusted to 80% of the field capacity in all pots after transfer of seedling, and the pots were irrigated every morning during the experiment to maintain 80% field capacity. The water was weighed and adjusted in ten randomly selected pots from each trial. To optimize irrigation and reduce plant growth effects, all pots were weighed and watered equally.

**Table 1:** Soil physicochemical properties.

Soil properties	Values
Soil texture	Sandy texture with loam
Sand	76.9%
Silt	12.0%
Clay	16.0%
pH	7.9
CEC	5.1 dS/m
ECe	0.9 dS/m
Carbonates	1.40 meq/L
Bicarbonates	3.0 meq/L
Organic matter	2.1%
Organic carbon	5.01%
Chlorides	2.09 meq/L

Plants (17 days old) were subjected to PEG 12% -induced drought stress for 1 week, followed by a subsequent 1-week recovery period till the morphological responses appeared differentially, while the control was irrigated with tap water. A total of eight different treatments were included in the experiment as mentioned below:

1. Control (CK) = no drought stress induction.
2. SAB = inoculated endophytic fungal isolate.
3. CBW = inoculated endophytic fungal isolate.
4. SAB and CBW = consortial inoculation of endophytic fungal isolates.
5. PEG 12% = drought stress induction.
6. PEG 12% + SAB = drought stress induction and inoculated endophytic fungal isolate.
7. PEG 12% + CBW = drought stress induction and inoculated endophytic fungal isolate.
8. PEG 12% + SAB + CBW = drought stress induction and consortial inoculation of endophytic fungal isolates.

Plants were incubated at light intensity (300 μmol m^−2^ s^−1^), day/night temperature (25–28/15–17°C), relative humidity (70/85%), and photoperiod (17/7 h) in controlled environmental conditions.

#### 2.6.3 Biochemical analysis

The chlorophyll contents in maize leaves were measured as described previously (Maclachlan and Zalik, 1963). Leaf samples were ground from 30-day-old plants using 80% acetone (3 mL). Total soluble sugars were estimated in ground fresh plant tissue (0.5 g), as mentioned by Mohammadkhani and Heidari (2008). Protein content was determined using fresh leaf tissues (1 g) following the method (Lowry et al., 1951). Total lipids were estimated using fresh leaf tissues (1 g) following the procedure (Vogel, 1956), while the proline contents were recorded according to Bates et al. (1973), using 2 g of fresh maize leaves. For the determination of flavonoid and total phenol contents, 1 g of fresh maize leaves was used. IAA quantification from maize leaves was carried out using the description of Benizri et al. (1998).

To estimate ABA content, the method used with 0.5 g leaf tissue powder, by adding a reaction buffer of 5 mL comprising of 2 N NH_4_OH, methanol, and chloroform (3,12,5 by volume), at pH 2.5, with extractions (3X) using 15 mL of ethyl acetate and 25 mL of dH_2_O. The chloroform phase was discarded after separation, as mentioned earlier (Ergün and Topcuoğlu, 2002). The pH was adjusted to 2.5 in the aqueous phase. To extract free ABA, ethyl acetate (15 mL) was used three times and incubated (70°C/1 h). Finally, elution with 2 mL of methanol followed by 45°C evaporation and absorption was recorded at 263 nm.

The 3,3-diaminobenidine (DAB) assay was performed following the method mentioned earlier (Thordal-Christensen et al., 1997) to detect the H_2_O_2_ accumulation. Approximately 1 cm of plant parts from the leaves of 30-day-old maize was vacuum-infiltrated with a DAB stainer and submerged in 90% ethanol (10 min, 70°C) to remove chlorophyll. Freshly prepared DAB solution was used to avoid auto-oxidation, and DAB polymerization spots of the H_2_O_2_ were recorded through photographs.

Catalase activity in maize leaves was quantified following the procedure mentioned previously (Chandlee and Scandalios, 1984). Fresh maize leaves (0.2 g) were crushed in 2 mL of phosphate buffer, and the centrifugation was done at 10,000 rpm for 5 min to separate the supernatant. Catalase enzyme activity was recorded at 240 nm at 30-s intervals (extinction coefficient = 0.036 mM/cm). The method described earlier (Asada, 1992) was employed for ascorbate peroxidase activity. A sample prepared from 0.2 g of tissue was mixed with the substrate solution added with the enzyme (0.1 mL). Optical density was assessed at 290 nm, and the optical density reduction was observed every 30 s for up to 7 min.

#### 2.6.4 Anatomical assessment for stem and stomatal

The stomatal anatomy was examined by the procedure mentioned earlier (Javed et al., 2022). To explore stomatal anatomy, leaf peels were floated in dH_2_O (2 h) under constant illumination and inspected at 100× magnification of a light microscope.

#### 2.6.5 RT-qPCR analysis for drought resistant marker gene expression analysis in maize

The total RNA was isolated from 30-day-old maize plants (root and shoot tissue) for RT-qPCR analysis as described earlier (Rahman et al., 2023). To generate the first strand of cDNA, 2 μg of pure RNA was used as the template using SuperScript II reverse transcriptase (Invitrogen, Lyon, France). The primer oligo (dT) synthesized cDNA. The normalization was done with the internal control *ZmActin2* as reported by Shyamli et al. (2021). Final RT-qPCR was performed on the ABI PRISM 7900HT, as described earlier (Rahman et al., 2023). For each treatment, three separate biological repeats were performed with at least three technical replicates. Gene sequences used for RT-qPCR analysis in the current study were retrieved from the MaizeGDB database.^2^ Primer sequences with gene accession numbers are given in Table 2.

**Table 2:** qPCR primers used for gene expression.

Gene name	Accession numbers	Primers sequences
*ZmActin2*	*Zm00001d013873*	*F-GCCATCCATGATCGGTATGG*
		*R-GTCGCACTTCATGATGGAGTTG*
*ZmBSK1*	*Zm00001d048345*	*F-ACCTCCATCCCGTGCTCTTG*
		*R-GGGTGTTGCGGTTGTGGAG*
*ZmcAPX*	*Zm00001d007234*	*F-TGAGCGACCAGGACATTG*
		*R-GAGGGCTTTGTCACTTGGT*
*ZmCAT1*	*Zm00001d014818*	*F-TGGAGGGCTTTGGTGTCAAT*
		*R-TAGATCCTTCGTCGCATGGC*

### 2.7 Statistical analysis

Experiment data were analyzed using GraphPad Prism 9.0.0 (121) software. SPSS V. 21.0 (SPSS, Chicago, IL, United States) was used to confirm statistical data by performing Duncan’s multiple range test mean separations. Multiple statistical bars showed significant differences (*p* < 0.05). Principal components analysis was performed for assessment of the effect of endophytes on plant parameters such as primary and secondary metabolites, antioxidants, and fresh and dry weight, under drought stress.

## 3 Results and discussion

### 3.1 Endophytic fungal isolation, characterization, and identification

Exploiting the beneficial and habitat-adapted microbes is a rather consistent and sustainable methodology for stress management and improving plant growth and yield. Several reports showed the benefits of habitat-adapted endophytes in plants against abiotic stresses including drought, such as *Trichoderma gamsii*, *Fusarium proliferatum*, *Microdochium majus*, *Aspergillus aculeatus*, and *Meyerozyma guilliermondii*, from xerophytic plant *Carthamus oxyacantha* induced drought stress tolerance in *Moringa oleifera* (Rehman et al., 2022; Javed et al., 2022). Desert-adapted fungal endophytes (*Aspergillus terreus*, *Aspergillus fumigatus*, *Talaromyces variabilis*, and *Talaromyces omanensis*) isolated from *Rhazya stricta*, are known to enhance the growth of tomato plants under drought induced by a 15% solution of PEG-6000 (Halo et al., 2020, 2023).

The current study also exposed the successful recruitment of drought-tolerant fungal endophytes from the root tissue of *Conyza bonariensis* plant grown in arid environments, as shown in Figure 1. Preliminary screening observations showed that only SAB and CBW ably produced biomass under 12% PEG-induced drought stress, hence selected as drought stress-resistant fungal endophytes (Figure 2A).

For the morphological identification, the visual features of the fungal isolates were observed (colony morphology, growth pattern, and reproductive structures), as shown in Figure 1A. Sequences showed the highest homology of SAB with *Aspergillus oryzae* (88%) and CBW with *Aspergillus fumigatus* (91%). Hence, the strains SAB and CBW were identified as with *Aspergillus oryzae* and *Aspergillus fumigatus*, respectively (Figures 1D,E). Moreover, the compatibility response of SAB and CBW that both strains showed proliferative growth over each other without inhibition in growth response (Figure 1C).

### 3.2 *In vitro* drought stress tolerance response of SAB and CBW upon PEG 12% supplementation

In the water-limited environment, the endophytes also exudate auxins, gibberellins, and abscisic acid or combinations to trigger a signaling cascade that induces the growth of plants and promotes drought resistance (Waqas et al., 2012; Qiang et al., 2019; Martínez-Arias et al., 2021; Naz et al., 2022; Rehman et al., 2022). Moreover, in addition to auxins and ABA, the fungi produce salicylic acid via chorismate. This shikimate pathway end-product is the basic source for SA biosynthesis in bacteria, plants, and fungi (Mishra and Baek, 2021).

In the current study, the PEG-mediated drought stress responses of SAB and CBW are shown in Figures 2A,B, where 12% of PEG-treated media exhibited well-sustained biomass production. In the present study, the assessment of the stress tolerance ability of the SAB and CBW revealed that growth-promoting and stress-alleviating metabolites and antioxidants were induced (Figures 2C–I).

The elevation in total soluble sugars was recorded in the culture filtrate of SAB supplemented with PEG 12% compared to control. Total soluble sugars quantification data revealed that SAB showed 45 μg/mL (control) and 295 μg/mL (drought stress). The isolate CBW also showed an increase in high-soluble sugar content production 51 μg/mL (control) and 275 μg/mL (drought stress) (Figure 2C).

The results for the quantification of SA (Figure 2D) showed that SAB produced 485 μg/mL (control), 1,047 μg/mL (drought stress), and CBW 222 μg/mL (control), 1,027 μg/mL (drought stress).

The results revealed that both the isolates produced IAA and SA (Figure 2E) under control as well as drought stress conditions. The isolate SAB produced IAA hormone 194 μg/mL (control) and 113 μg/mL (drought stress), and the isolate CBW produced IAA 169 μg/mL (control) and 106 μg/mL (drought stress).

The production of flavonoid (Figure 2F) is shown upgraded significantly (*p ≤* 0.05) by PEG supplementation to SAB showing 1.02 μg/mL (control) and 1.64 μg/mL (drought stress) and CBW 0.75 μg/mL (control) and 1.54 μg/mL (drought stress).

Differentially sufficient concentrations of phenolic (Figure 2G) were produced by isolates of SAB and CBW under control as well as drought stress conditions. SAB during supplementation of PEG 12% 485 μg/mL (control) and 222 μg/mL (drought stress), and CBW produced 438 μg/mL (control), and 215 μg/mL (drought stress).

Quantification of proline (Figure 2H) showed that SAB produced 0.64 μg/mL (drought stress) and 0.45 μg/mL (control) and CBW produced 0.52 μg/mL (control) and 0.62 μg/mL (drought stress). After the stress tolerance assessment of isolates against PEG 12% induced drought, antioxidant activity APX (Figure 2I) was also recorded from the culture filtrate of SAB and CBW. Compared to the control, isolates during drought stress conditions showed a high antioxidant enzymatic activity. SAB showed 1.5 Units/g FW (drought stress) and 0.5 Units/g FW (control), and CBW showed 1.2 Units/g FW (drought stress) and 0.4 Units/g FW (control).

### 3.3 Effect of SAB and CBW on vegetative growth attributes of maize under drought stress

Plant roots absorb water and minerals from the soil, accumulate photosynthetic products, perform nitrogen fixation in legumes, and synthesize metabolites. Internal factors, such as hormones (auxins, ABA, brassinosteroids, and so on), and external environmental factors influence the root function (Zhang et al., 2022).

Multiple studies show that endophyte enrichment boosts root biomass and plant development during drought by promoting the cumulative absorption of nutrients including phosphorus and zinc may protect photosynthetic machinery and enhance photosynthesis activity. This further strengthens back the impact of fungal endophytes on the development and growth of plants activated by the release of phytohormones and bioactive chemicals, or inadvertently by optimizing the expressions of host genes and physiological responses, which eventually modulates water uptake, conduction, and conservation, and activates cellular tolerance to stress (Saddique et al., 2018; Li et al., 2019; Nataraja et al., 2022).

Researchers have also found that osmotic stress-tolerant *Entomophaga chlamydospora*, *Cladosporium oxysporum*, and *Paraphoma* sp., isolated from *Helianthemum scoparium-*induced drought stress tolerance in the host plant (*Helianthemum scoparium*) as well as non-host (*Glycyrrhiza uralensis* and *Zea mays*) plants after beneficial association and promoted the root growth (Li et al., 2019). Similarly, isolated endophytes *Pseudomonas terricola* and *Acaulospora chlamydospore* under drought stress proficiently promoted the growth and bioactive content of licorice plants (He et al., 2021). *Ormosia hosiei* showed a drought tolerance response by a dark septate endophyte that modifies root shape and architecture by phytohormonal modulation (Liu and Wei, 2019).

As a long-chain polymer, PEG induces drought stress in plants due to its presence in a diverse spectrum of molecular weights. To assess the drought resistance of crop, PEG is used by researchers during seedling growth as it prevents seed germination by dropping water potential, and compared to main roots, the more noticeable effects occur on shoots. Based on germination indices *in vitro* screening for selecting drought-tolerant genotypes, one of the most accurate procedures is the use of PEG (Mahpara et al., 2022).

**Figure 3:**
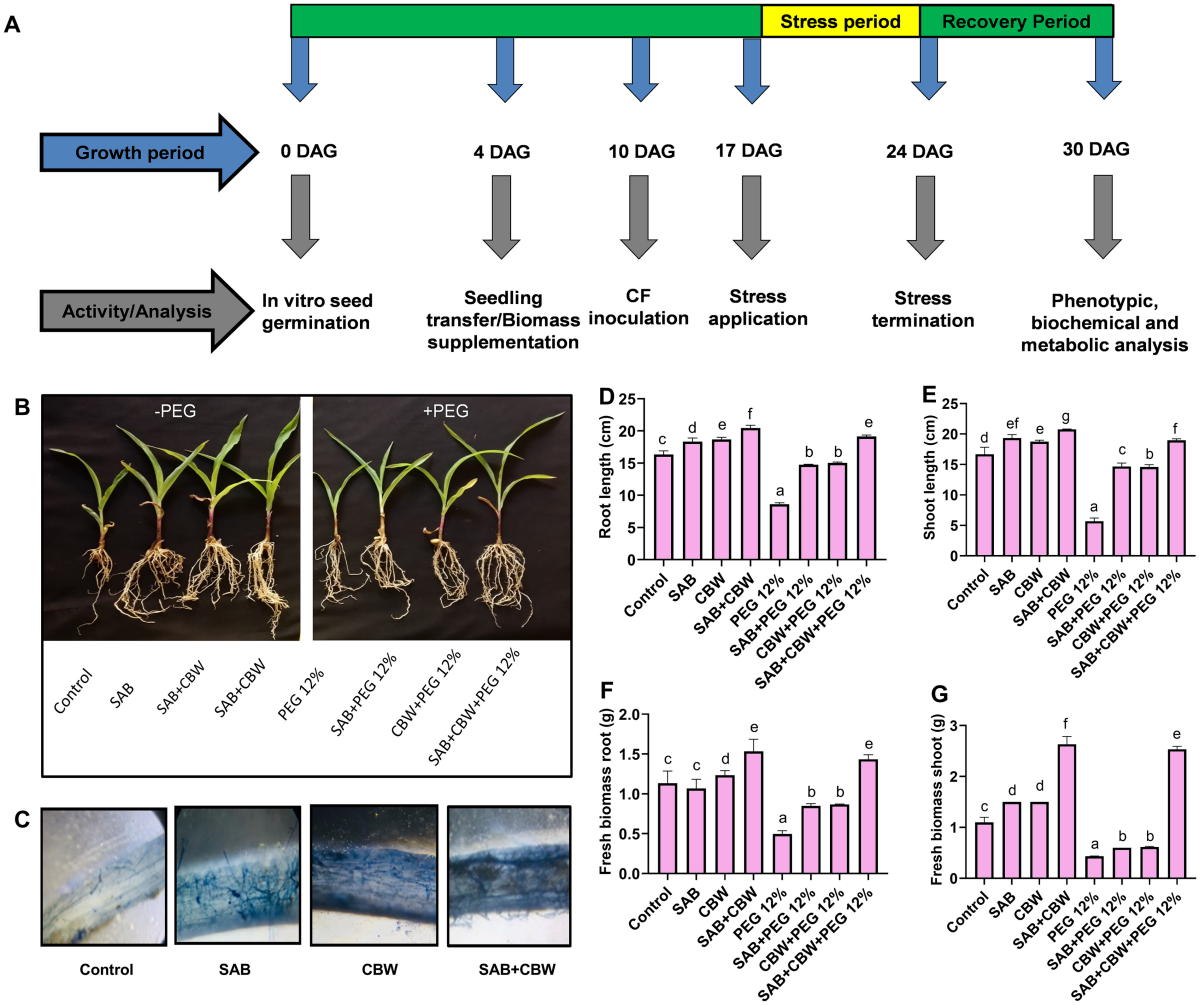
Influence of the fungal isolates SAB and CBW on maize plant growth under drought stress. **(A)** Graphic illustration showing bioassay indicated by arrows at various time-course activities, **(B)** phenotypes of 30-day-old seedlings grown without and with PEG-induced drought stress, inoculated and non-inoculated with the SAB and CBW isolates, **(C)** microscopic view of maize root colonization by SAB and CBW, **(D)** root length, **(E)** shoot length, **(F)** root fresh weight, and **(G)** shoot fresh weight. Numerical data signify means ± SE presented as biologically distinct replicates with significant letters at *p ≤* 0.05 via a Duncan multiple range test (DMRT).

The influence of SAB and CBW on maize was explored under drought stress compared to the control in terms of shoot, root length, and fresh and dry weight (Figure 3). Root colonization of SAB and CBW (Figure 3C) was microscopically visualized showing effective colonization of fungi with maize.

Under PEG 12% induced drought stress, the isolate SAB and CBW significantly promoted maize root length with 14.7 cm (SAB), 14.9 cm (CBW), and 19.4 cm (SAB/CBW consortium), compared with PEG-induced drought-stressed plants with 8.6 cm root length after recovery period (Figure 3D), while root length with 14.0 cm (SAB), 14.5 cm (CBW), and 18.7 cm (SAB/CBW consortium), compared with PEG-induced drought-stressed plants with 5.6 cm root length after recovery period (Figure 3E).

Under PEG 12% induced drought stress, the isolate SAB and CBW significantly promoted maize root fresh weight with 0.83 g (SAB), 0.87 g (CBW), and 1.51 g (SAB/CBW consortium), compared with PEG-induced drought-stressed plants with 0.53 root fresh weight after recovery period (Figure 3F), while shoot fresh weight with 0.63 g (SAB), 0.62 g (CBW), and 2.60 g (SAB/CBW consortium), compared with PEG-induced drought-stressed plants with 0.43 g shoot fresh weight after recovery period (Figure 3G).

Under drought stress of PEG 12%, the widespread colonization and strong association of SAB and CBW proposed the interaction of maize with these isolates may have encouraged the maize growth via accelerated length of root and shoot as well as biomass and dry weight by providing stress-alleviating and growth-promoting metabolites.

Previously, fungal endophytes are known to improve growth by producing biomass and growth-related phytohormones upon stresses. In the present study, the widespread colonization and strong association of SAB and CBW under drought stress proposed the interaction of maize with these isolates has encouraged the host plant growth through hormonal and metabolic reprogramming. In *Pseudomonas tabulaeformis*, seedling development is boosted by the endophytic strain *Phoma* sp. (Zhou et al., 2021). Similarly, previous reports show that drought stress is induced in *Lolium perenne and Festuca arundinacea* by endophyte *Epichloe* (Decunta et al., 2021) with this in *Colletotrichum quintensis* the drought tolerance is induced by consortia of *Pseudocercospora Phaeosphaeria*, *Penicillium brevicompactum*, *Penicillium chrysogenum*, and *Enterobacter osmophilum*, and *Alternaria* sp. (Hereme et al., 2020).

### 3.4 Impact of SAB and CBW on the photosynthetic pigmentation of maize drought stress

Photosynthetic regulations are other primary plant strategies under drought conditions (Bakhshi et al., 2023). Drought impairs the physiological activities of plants such as photosynthesis (Ahluwalia et al., 2021). Under drought stress, photosynthetic performance is increased in Perennial ryegrass (*Lolium perenne*) inoculated with *Aspergillus aculeatus* compared to the control (Li et al., 2021).

**Figure 4:**
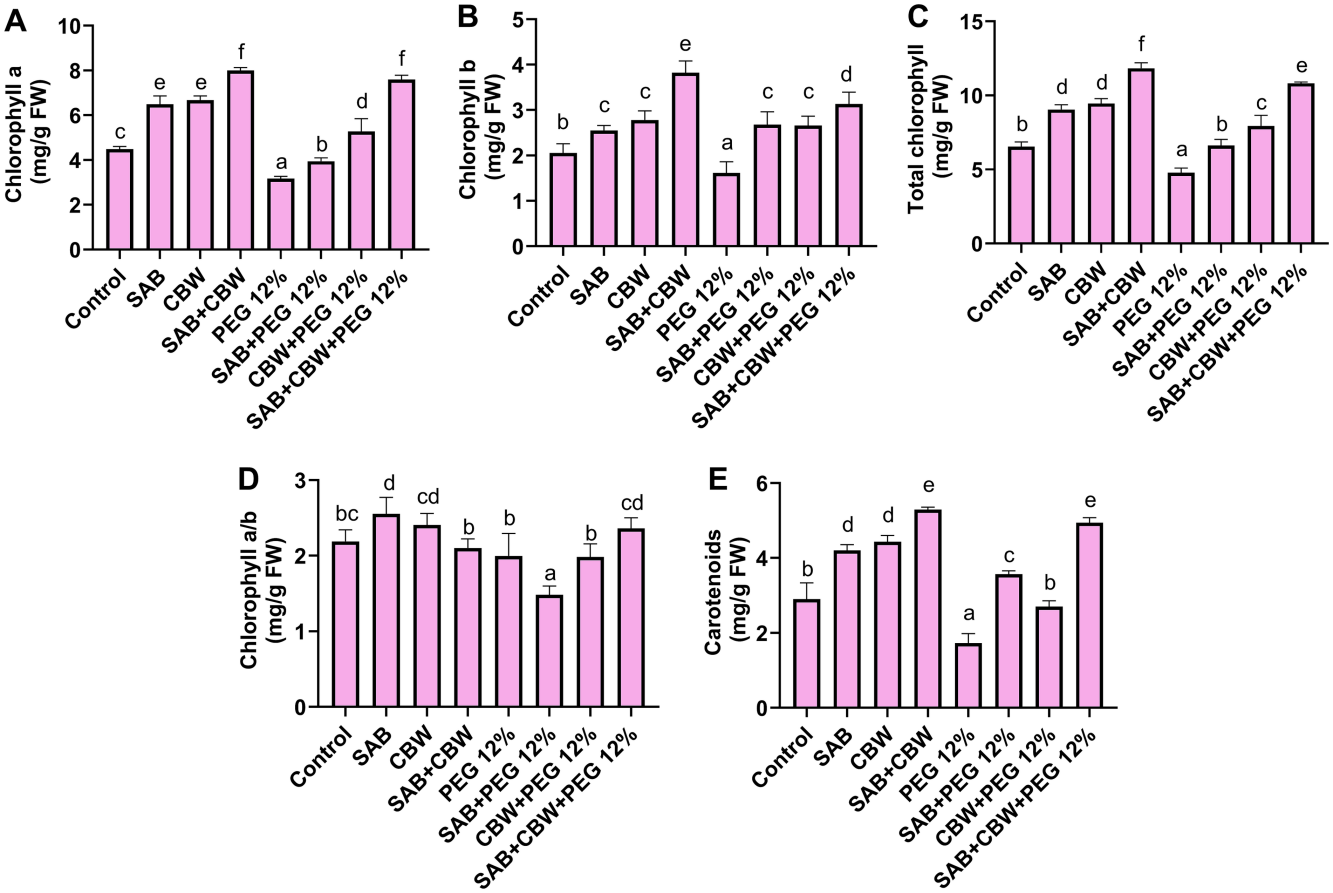
Effects of SAB and CBW on photosynthetic pigments of maize plants under drought stress. **(A)** Chlorophyll a, **(B)** chlorophyll b, **(C)** total chlorophyll, **(D)** chlorophyll a/b, and **(E)** carotenoids. Numerical data signify means ± SE presented as biologically distinct replicates with significant letters at *p ≤* 0.05 via a Duncan multiple range test (DMRT).

The photosynthetic potential of maize inoculated with SAB and CBW was assessed under drought stress induced by PEG. Under PEG 12% induced drought stress, the isolate SAB and CBW significantly promoted maize chlorophyll a with 4.06 mg/g FW (SAB), 5.5 mg/g FW (CBW), and 7.5 mg/g FW (SAB/CBW consortium), compared with PEG-induced drought-stressed plants with 3.07 mg/g FW after recovery period (Figure 4A), chlorophyll b with 2.3 mg/g FW (SAB), 2.5 mg/g FW (CBW), and 3.6 mg/g FW (SAB/CBW consortium), compared with PEG-induced drought-stressed plants with 1.07 mg/g FW after recovery period (Figure 4B), while total chlorophyll and chlorophyll a/b ration was also increased upon endophytic inoculated under drought stress in maize (Figures 4C,D). The carotenoid content was 3.6 mg/g FW (SAB), 2.6 mg/g FW (CBW), and 5.06 mg/g FW (SAB/CBW consortium), compared with PEG-induced drought-stressed plants with 1.9 mg/g FW after the recovery period (Figure 4E).

### 3.5 Effects of SAB and CBW on the production of osmolytes and metabolites of maize under drought stress

Plants synthesize several metabolites and osmolytes to withstand drought, and plant stress tolerance response is partially reflected in the production and accumulation of proline, total soluble sugars, protein, and lipid content, glycine betaine, and organic acids (Seema et al., 2023). Plants accumulate proline as a result of stress conditions, which are contingent upon fluctuations in the activity of 1-pyrroline-5-carboxylic acid synthase (P5CS), a critical enzyme in proline biosynthesis. Endophytes also stimulate the accumulation of suitable solutes such as proline, glycine betaine, soluble carbohydrates, and organic acids (Nagabhyru et al., 2013) contribute to drought tolerance by reducing osmotic potential and turgor maintenance. Moreover, enhanced proline synthesis during drought is reported by activating the biosynthesis pathway of proline through gene P5CS expression in the modulated by endophytes (Saddique et al., 2018). Ghaffari et al. (2019) reported that *Piriformospora indica* colonization regulates plant metabolites and maintains the presence of aquaporins in drought-stressed plants. Endophytic fungus, *cladosporioides*, and an unknown species of *Ascomycota*, isolated from tobacco, increased drought tolerance through osmolytes and antioxidants (Dastogeer et al., 2018). Moghaddam et al. (2021) reported fungal endophyte *Pseudomonas macrospinosa* induces drought tolerance in tomatoes and cucumbers by increasing proline and antioxidants.

**Figure 5:**
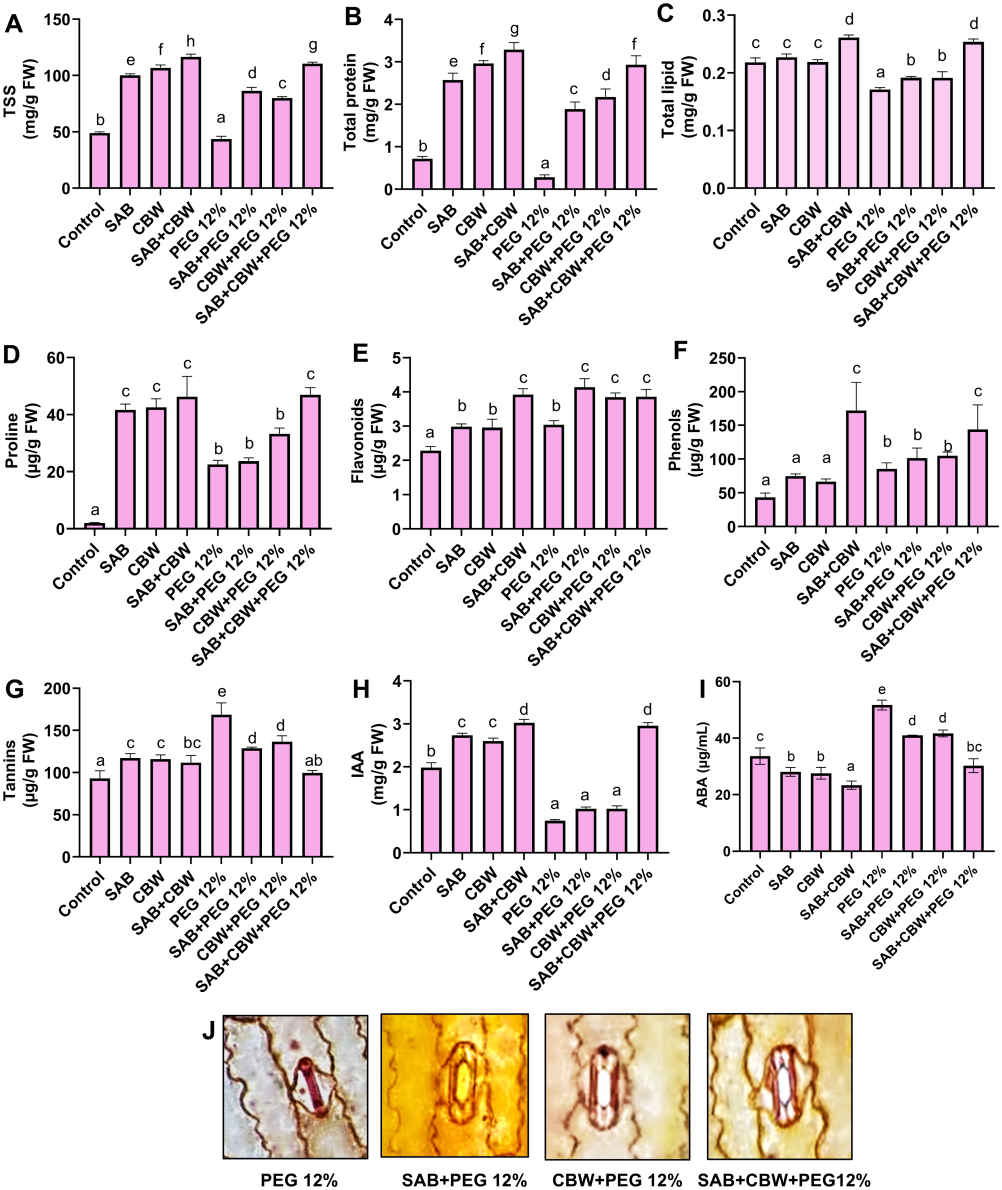
Effects of SAB and CBW on metabolic and hormonal contents of maize plants under drought stress. **(A)** Total soluble sugars, **(B)** protein, **(C)** total lipids, **(D)** proline, **(E)** flavonoids, **(F)** phenolics, **(G)** tannins, **(H)** IAA, **(I)** ABA, and **(J)** stomatal anatomy. Numerical data signify means ± SE presented as biologically distinct replicates with significant letters at *p ≤* 0.05 via a Duncan multiple range test (DMRT).

In the present investigation, the metabolic production of maize inoculated with SAB and CBW was assessed under PEG-mediated drought stress (Figure 5). Under PEG 12% induced drought stress, the isolate SAB and CBW significantly promoted the total soluble sugars by 87 mg/g FW (SAB), 79 mg/g FW (CBW), and 109 mg/g FW (SAB/CBW consortium), compared with PEG-induced drought-stressed plants by 46 mg/g FW after recovery period (Figure 5A).

The protein content of 1.85 mg/g FW (SAB), 2.5 mg/g FW (CBW), and 2.9 mg/g FW (SAB/CBW consortium), compared with PEG-induced drought-stressed plants by 0.23 mg/g FW after the recovery period (Figure 5B). The lipid content by 0.19 (SAB), 0.2 (CBW), and 0.25 (SAB/CBW consortium), compared with PEG-induced drought-stressed plants by 0.16 mg/g FW after the recovery period (Figure 5C). The proline content by 24 (SAB), 32 (CBW), and 46 (SAB/CBW consortium), compared with PEG-induced drought-stressed plants with 20 mg/g FW after the recovery period (Figure 5D).

In addition to primary metabolites and osmolytes, several studies have shown that under drought plants also increase the secondary metabolites (polyphenols, flavonoids, and tannins) by recognizing the internal signal through metabolic indications and external ecological variations to help against stress via antioxidant activity. Under abiotic and biotic stress, flavonoids, particularly iso-flavonoids, perform a significant role. Tannins (polymeric flavanols: flavan-3-ols and flavan-3-4-diols) are known for their ability as antioxidants to alleviate oxidative stress in poplar under drought and UV-B stress (Gourlay et al., 2022). Present study exhibited that the maize inoculated with SAB and CBW under drought enhanced the flavonoid, phenols, and tannin contents compared to the control.

The flavonoid content was 4.1 (SAB), 3.9 (CBW), and 4.06 (SAB/CBW consortium), compared with PEG-induced drought-stressed plants by 2.8 mg/g FW after the recovery period (Figure 5E). The content of phenols by 118 (SAB), 109 (CBW), and 130 (SAB/CBW consortium), compared with PEG-induced drought-stressed plants with 94 mg/g FW after the recovery period (Figure 5F). Total tannins were reduced by 128 (SAB), 132 (CBW), and 96 (SAB/CBW consortium), compared with PEG-induced drought-stressed plants by 183 mg/g FW after the recovery period (Figure 5G).

### 3.6 Effects of SAB and CBW on the hormonal contents and stomatal activity of maize under drought stress

In addition to photosynthetic activity, osmolytes, and metabolic production, to survive under drought stress, the turgor pressure, conductance activity of stomata, and plant membrane stabilization are also crucial. Similarly, by adjusting plant hormones, metabolites, antioxidant systems, and scavenging ROS, *Fusarium proliferatum* obtained from the roots of *Rhazya stricta* increases the drought resistance of sunflowers (Seema et al., 2023). Several endophytes activate the biosynthesis of auxin in the host, which leads to the alteration of the architecture of the root by affecting the length number, volume, and biomass of the roots, thus increasing the ability of water uptake, conduction, and translocation during droughts. Several endophytes are explored with the ability of auxin production that promotes the growth of roots in plants under drought stress (Nataraja et al., 2022).

During prolonged drought, maize reduces the stomatal aperture and increases the density, which hinders the photosynthetic activity by reduction in transpiration, which threatens plant growth and survival (Serna, 2022). Salicaceae endophytes modulated the stomatal behavior and increased water use efficiency in rice (Rho et al., 2018). Abscisic acid (ABA) released by endophytes also elicited the plants to initiate a signaling cascade for drought tolerance induction in their hosts. Several additional substances influence drought responses by raising the concentration of ABA in the host. Elevated ABA levels accelerate sensibility, reduce lateral root growth, and lengthen main roots—all of which are essential for improving water uptake—while also stimulating stomatal closure through the generation of H_2_O_2_ to maintain moisture. Several reports showing the endophyte association correlates with ABA levels, negatively, positively, and neutrally (Watts et al., 2023).

In conjunction to the previous reports, the present study and the findings also revealed that IAA was increased by 1.1 mg/g FW (SAB), 1.3 mg/g FW (CBW), and 3.1 mg/g FW (SAB/CBW consortium), compared with PEG-induced drought-stressed plants by 0.71 mg/g FW after the recovery period (Figure 5H); however, ABA content was reduced by 40 mg/g FW (SAB), 41 mg/g FW (CBW), and 29 mg/g FW (SAB/CBW consortium), compared with PEG-induced drought-stressed plants by 50 mg/g FW after the recovery period (Figure 5I). In addition, the influence of SAB and CBW isolates was also observed on the stomatal anatomy of maize under PEG-mediated drought stress. The inoculation of SAB and CBW isolates (individual as well as a combination) exhibited an optimal activation in stomatal opening under PEG-mediated drought stress compared with the maize plants under drought stress showing stomatal closing (Figure 5J).

### 3.7 Effects of SAB and CBW on the antioxidant potential of maize under drought stress

Scavenging the ROS by antioxidants further aids the resistance mechanisms in plants that upsurge the growth under stress. Therefore, several plant species evolved strategies to defend themselves against stress damage over time. The enzymatic antioxidants, including superoxide dismutase, ascorbate peroxidase, catalase, and glutathione reductase, scavenge ROS under drought stress while shielding plants from oxidative damage. Furthermore, many studies show that endophytes play a significant role in imparting resistance to drought by activating a variety of cellular tolerance features. Increased capability for detoxifying ROS under drought reduces the damage to cellular membrane damages and macromolecule degradation. For example, the stomata behavior and ROS-scavenging systems are modulated by *Piriformospora indica* symbiotic association in rice under drought stress (Tsai et al., 2020). In addition to this, the consortia of *Microdochium majus*, *Aspergillus aculeatus*, and *Meyerozyma guilliermondii* enhanced growth features, increased photosynthetic pigments, growth, and stress-related phytohormones, and antioxidant enzymes, and reduced ROS in *Moringa oleifera* under drought stress (Javed et al., 2022).

**Figure 6:**
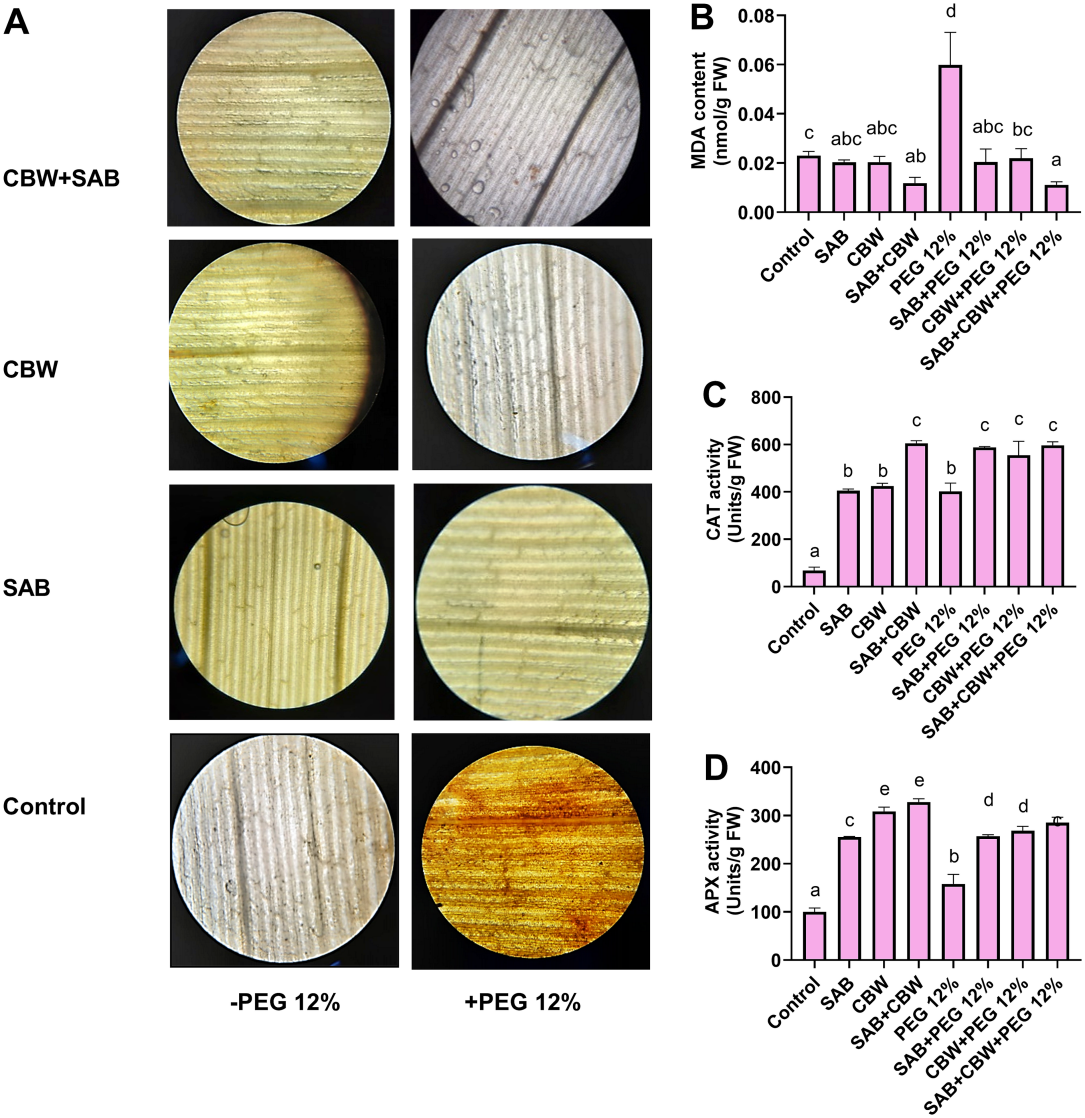
Effect of SAB and CBW inoculation on maize antioxidant potential under drought stress. **(A)** DAB staining, **(B)** content of MDA, **(C)** catalase enzyme activity, and **(D)** ascorbate peroxidase enzyme activity. Numerical data signify means ± SE presented as biologically distinct replicates with significant letters at *p ≤* 0.05 via a Duncan multiple range test (DMRT).

In the current investigation, the oxidative damage in terms of ROS generation in maize plants was investigated in response to drought stress. Using DAB (3,3′-diaminobenzidine) staining, the histochemical experiment demonstrated increased H_2_O_2_ generation and accumulation in the leaves of maize plants. However, the DAB staining was reduced in maize leaf tissues of the plants inoculated with SAB and CBW, upon drought stress in comparison with the control (Figure 6A). Furthermore, the MDA content was decreased by 0.014 nmol/g FW (SAB), 1.3 nmol/g FW (CBW), and 3.1 nmol/g FW (SAB/CBW consortium), compared with PEG-induced drought-stressed plants by 0.71 nmol/g FW after the recovery period (Figure 6B); however, catalase enzyme activity was increased by 592 Units/g FW (SAB), 570 Units/g FW (CBW), and 612 Units/g FW (SAB/CBW consortium), compared with PEG-induced drought-stressed plants by 442 Units/g FW after the recovery period (Figure 6C); and ascorbate peroxidase activity was increased by 260 Units/g FW (SAB), 275 Units/g FW (CBW), and 292 Units/g FW (SAB/CBW consortium), compared with PEG-induced drought-stressed plants by 171 Units/g FW after the recovery period (Figure 6D).

### 3.8 Multivariant evaluation using principal component analysis (PCA)

**Figure 7:**
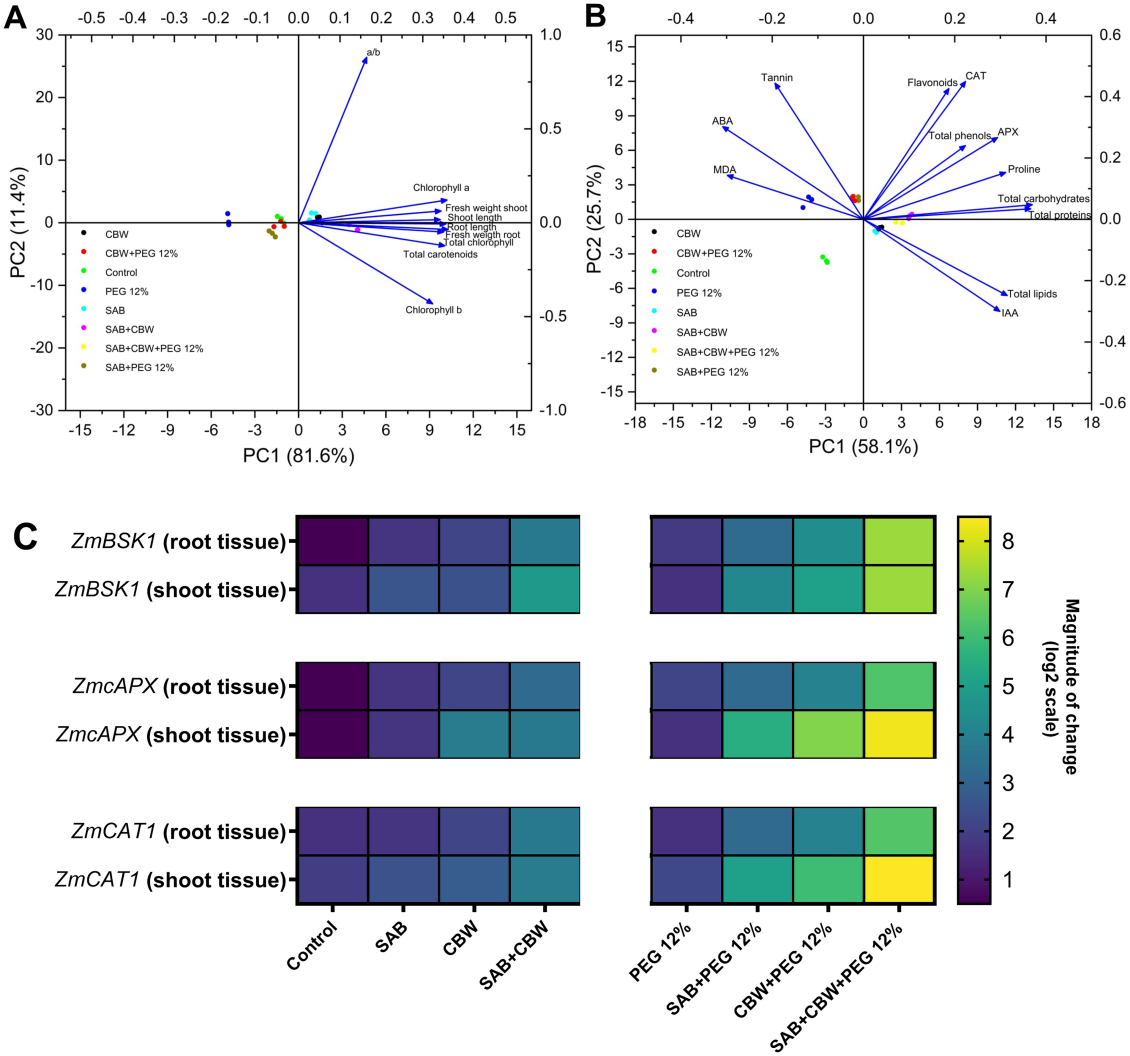
Multivariant assessment and gene expression analysis. **(A)** Principal component analysis of growth attributes comprising growth and physiological attributes comprising chlorophyll a, and b, total chlorophyll, chlorophyll a/b ratio, and carotenoid content; **(B)** Principal component analysis of metabolic, hormonal, and antioxidant parameters comprising phenols, proteins, flavonoids, sugars, proline, lipids, tannins, MDA content, IAA, ABA, ascorbate peroxidase, catalase activity of maize under drought stress induced by PEG, inoculated with several endophytic treatments: SAB and CBW, alone or in combination, compared with the control; and **(C)** *ZmBSK1*, *ZmAPX* and *ZmCAT1* gene expression measured by RT-qPCR in root and shoot tissues. Numerical data signify means ± SE presented as biologically distinct replicates with significant letters at *p ≤* 0.05 via a Duncan multiple range test (DMRT).

Endophytes have been found to reprogram the metabolome (Dhanyalakshmi et al., 2019), resulting in the activation of multiple and complex, stress-adaptive traits in host plants. In the current study, under PEG-mediated drought stress, to acquire a comprehensive understanding of adaptability concerning stress-adaptive traits of maize by various endophytic associations (SAB and CBW), a principal component analysis (PCA) was performed on all recorded growth, physiological, antioxidant, and metabolic parameters (Figures 7A,B).

PCA ordination plot was prepared to present the cumulative evaluation of the concerted information revealing the association between maize plants and endophytes under PEG 12% induced drought stress. The PCA regarding the growth promotion of maize associated with endophytes SAB and CBW (Figure 7A) explained 93% of the total variance (81.6% in PC1 and 11.4% in PC2). The plot showed a clear segregation of treatments and variables in different quadrants. The upper left quadrant of the negative side of PC1 (Q1) included control and PEG 12% treatment. A second group clustered on the positive side of PC1 (Q2) comprising of SAB, CBW, and SAB + CBW + PEG 12% presenting higher chlorophyll a/b ratio, fresh weight shoot, and shoot length. While the lower left (Q3) quadrant illustrates SAB + PEG 12% and CBW + PEG 12%. Finally, the Q4 is CBW + SAB with the highest root length, fresh weight root, total chlorophyll, total carotenoids, and chlorophyll b.

In addition to this, the PCA plot prepared for the evaluation of information regarding the metabolites of maize (Figure 7B) estimated during PEG 12% induced drought showed total variance of 83.8% being (PC1 25.7% and PC1 58.1%) the upper left quadrant that is Q1 showed the highest MDA, ABA, and tannins and comprising the treatments PEG 12%, CBW + PEG 12%, and SAB + PEG 12%. The second quadrant (Q2) depicted SAB + CBW presenting the highest level of flavonoid, CAT, APX, total phenols, proline, total carbohydrates, and total protein. The lower left quadrant showed the control, and the last quadrant Q4 comprises SAB, CBW, and SAB + CBW + PEG 12% with higher total chlorophyll, total carotenoids, total lipids, and IAA.

### 3.9 Stress-resistant marker gene expression in maize plants under drought stress inoculated with SAB and CBW

Another significant mechanism driving endophyte-mediated drought tolerance is the activation of drought-responsive gene transcription in the host (Sherameti et al., 2008), to reprogram the transcriptome and proteome, resulting in the activation of multiple and complex, stress-adaptive traits in host plants (Sampangi-Ramaiah et al., 2020). For instance, in *Moringa oleifera* L. under drought stress the consortia of *Microdochium majus*, *Aspergillus aculeatus*, *and Meyerozyma guilliermondii* upregulated the expression of genes (*MolHSF3*, *MolAPX*, and *MolHSF19*) indicates that fungal endophytes have a significant regulatory role in alleviating drought stress at the molecular level (Javed et al., 2022). Moreover, *Trichoderma gamsii* and *Fusarium proliferatum* endophytic fungal isolates from the xerophytic plant *Carthamus oxyacantha* modulated the expression of ethylene biosynthesis/signaling-related genes, and antioxidant enzymatic genes via ethylene metabolism in *Moringa oleifera* under drought stress (Rehman et al., 2022), *P. indica confers drought tolerance in maize* by elevating the antioxidant potential and gene expression level of drought-related genes. In addition to this, the transcript abundance of the inherent stress-responsive genes such as *DREB2A*, *ANAC072*, *ERD1*, *CIPK3*, *CBL1*, *PLDδ*, and *HAT*, which are functionally associated with a variety of cell signaling, defense, and metabolic mechanisms, is also substantially modulated by the endophytes in maize under drought stress (Xu et al., 2017). The induction of the proline biosynthetic pathway gene, P5CS, in the presence of an endophyte is another example that contributes to the increased synthesis of proline during drought (Saddique et al., 2018). Moreover, the expression of the wax biosynthetic pathway genes (e.g., *KAR*, *FabG*, *FAR*, *fadD*, *desB*, *SSI2*, *KCS*, *BiP*, and *ABCB1*) was substantially altered by endophyte inoculation during drought, which led to a change in the wax composition of the host (Zhao et al., 2022).

In plants, brassinosteroids are phytohormones (polyhydroxylated steroids) that contribute to regulating the physiological mechanisms of plants. Brassinosteroids are plant hormones that promote root growth and modulate plant height. Furthermore, BRs have the potential to induce plant tolerance to a diverse array of biological and abiotic stresses, including oxidative stress, pathogen infection, temperature stress, salinity stress, temperature stress, and drought stress (Sun et al., 2020). Previously, the brassinosteroid-signaling kinase 1 (ZmBSK1) from maize was identified as an interaction protein of ZmCCaMK. BSK1 is a critical component of the BR signaling pathway (Nolan et al., 2020). Recently, it has been demonstrated that ZmBSK1-mediated phosphorylation of ZmCCaMK plays a significant role in regulating the tolerance of maize plants to drought and salt stress (Liu et al., 2021, 2022).

The present study exposed the modulatory effect of SAB and CBW endophytic fungi on the gene expression and regulation via the upregulation of *brassinosteroid-signaling kinase 1 (ZmBSK1)* modulating brassinosteroid-signaling pathway. In addition to this, under drought stress the antioxidant enzymatic gene expression of the *cytosolic APX isoenzyme (ZmcAPX)*, and *catalase1 enzyme (ZmCAT1)* (Figure 7C) were also induced by SAB and CBW elevating the drought tolerance by producing the ROS-scavenging enzymes that corroborated the enhanced tolerance to PEG-induced drought stress in maize, via optimizing the ROS levels and thereby reducing oxidative damage.

## 4 Conclusion

The present research accomplishes with focuses on the possible function of habitat-adapted fungal endophytes SAB and CBW cultivated maize resilience to drought by enhancing the growth, and stress tolerance, via brassinosteroid regulation, metabolic induction, osmolytes elevation, antioxidant activation, and stomatal activity optimization, as depicted in the graphical abstract. In dry and xeric conditions, the current study suggests adopting SAB and CBW fungal endophytes as biostimulators with great antioxidant potential to activate plant growth and alleviate drought stress, particularly in desert regions. Additionally, in maize, PCA and molecular analysis demonstrate the signaling and interactions between fungus and plant, which have illuminated the role of *ZmBSK1*, *ZmcAPX*, and *ZmCAT1* gene regulatory cascade triggered upon drought and inducing the resistance by enhancing the antioxidant potential of maize under drought stress via stress-related biomolecules including brassinosteroids regulation. Therefore, the current research refers to the current findings as beneficial for researchers, farmers, and agro-industry scientists for exploiting the consortium of habitat-adapted endophytic fungi as biostimulators for mitigating the drought stress in maize for sustainable yield in arid and semiarid regions of the world.

## Data Availability

The datasets presented in this study can be found in online repositories. The names of the repository/repositories and accession number(s) can be found at: https://www.ncbi.nlm.nih.gov/genbank/, PP892795, PP859235.

## Footnotes

^1^http://blast.ncbi.nlm.nih.gov/Blast

^2^https://www.maizegdb.org/
